# Aripiprazole augmentation in poor insight obsessive-compulsive disorder: a case report

**DOI:** 10.1186/1744-859X-7-26

**Published:** 2008-12-23

**Authors:** Michele Fornaro, Filippo Gabrielli, Chiara Mattei, Valentina Vinciguerra, Pantaleo Fornaro

**Affiliations:** 1 Dipartimento di Neuroscienze, Oftalmologia e Genetica (DINOG), Sezione di Psichiatria, Università di Genova, Italy

## Abstract

**Background:**

Obsessive-compulsive disorder is associated with a relevant impairment in social and interpersonal functioning and severe disability. This seems to be particularly true for the poor insight subtype, characterised by a lack of consciousness of illness and, consequently, compliance with treatment. Poor responsiveness to serotonergic drugs in poor insight obsessive-compulsive patients may also require an augmentation therapy with atypical antipsychotics.

**Methods:**

We reviewed a case in which a patient with a long history of poor insight obsessive-compulsive disorder was treated with a high dosage of serotonin reuptake inhibitors.

**Results:**

The treatment resulted in a poor outcome. This patient was therefore augmentated with aripiprazole.

**Conclusion:**

Doctors should consider aripiprazole as a possible augmentation strategy for serotonergic poor responder obsessive-compulsive patients, but further research on these subjects is needed.

## Background

Despite the fact atypical antipsychotics (AA) represent a relatively novel pharmacological class, they are widely prescribed by an increasing number of clinicians for heterogeneous conditions. Using AA augmentation therapy for serotonin reuptake inhibitors (SRIs) refractory obsessive-compulsive disorder (OCD) with poor insight (PI) is just one of such recently proposed clinical uses for this class of drugs [1]. Although augmentation strategies for OCD are quite often used by clinicians, literature data are generally limited to some case reports or preliminary studies.

This lack of evidence seems even more relevant for specific yet quite common OCD conditions, such as PI, reported to be as prevalent as 15% to 36% all OCD cases [2], usually showing harder to treat features [3] and association with a greater impairment in quality of life (QOL) [4].

## Methods

The patient is a 34-year-old female with a severe PI-OCD, first diagnosed 8 years ago. She was referred by her mother to our outpatients department for worsening of OCD and a progressive lack of insight started 3 years ago. She had no other relevant medical or psychiatric comorbidities and no history for full-threshold OCD spectrum disorders. For the first 4 years of treatment, she received paroxetine 40 mg/day, showing poor response. When admitted to the outpatients department, her obsessions were mainly about being physically sick and bodily contaminated, with washing and cleaning compulsions. Additionally, she showed intense fear of developing severe side effects to the suggested therapy, resulting in belching compulsions before taking medications.

Insight into her illness and therefore compliance to the therapy was poor. In such scenarios, obtaining the patient's compliance to medication was as important as it was difficult to acheive. Because of her initial belching compulsion, it required about 2 months to start a pharmacological therapy. The patient was asked to answer the Yale-Brown Obsessive-compulsive Scale (Y-BOCS) and the Brown Assessment of Beliefs Scale (BABS) when a maximum label dose therapy with sertraline at 200 mg/day (100 mg twice a day) was started.

## Results

The 2 submitted scales showed total scores of 50 and 23, respectively, (with a score of 4 for the item 'conviction' and 4 for the item 'insight' on the BABS); on the BABS scale, 4 is the highest possible score and it indicates worse sympthomatology [5,6].

A clinical follow-up was obtained after 1 month. The patient was asked to answer the same rating scales again. The new Y-BOCS total score was 47 while the BABS total score was recorded at 22 ('conviction' and 'insight' items were both scored as 4). Clinical improvement was poor (the patient still showed avoidant behaviour and reluctance to the pharmacotherapy). At this point, aripiprazole 10 mg/daily (in the morning) was introduced as augmentation strategy.

The second clinical follow-up was obtained 1 month later. The Y-BOCS total score was now 40 and the BABS total score was 19 (the 'conviction' and the 'insight' items were scored as 3). At this point the patient showed mild clinical improvement (same obsessions and compulsions were still present, but less intrusive).

At this point, an alternative therapy with clomipramine was also considered, but it was discarded because of a positive anamnesis for Sjogren syndrome, with xerostomia as a possible exacerbating factor due to the anticholinergic side effect of the considered drug.

The last clinical follow-up was obtained at the 120th day from the beginning of the therapy. The Y-BOCS total score was 32 and the BABS total score was 17 (the 'conviction' and the 'insight' items scored 4 – one more than previously). At this point the patient showed a further mild clinical improvement, requiring a longer follow-up.

## Conclusion

Obsessions and delusions have been traditionally viewed as dichotomous phenomena, with obsessions been defined as 'intrusive, ego-dystonic thoughts with the patient maintaining insight'.

By contrast, delusions have been defined as false beliefs held firmly by the patient without insight into the irrationality of the belief. However, obsessions and delusions may be better conceptualised as existing on a continuum of insight that ranges from good insight (overvalued ideation, as in typical OCD) to poor insight/no insight (delusional thinking, as in severe PI-OCD). Such a continuum of insight may be present among a variety of psychiatric disorders, such as OCD (including the PI subtype), dysmorphic disorder, hypochondriasis, eating disorders and psychotic disorders, such as schizophrenia and delusional disorder [6].

Psychiatric disorders sharing the 'psychotic' feature have been traditionally effectively treated using typical antipsychotics (AT). More recently clinicians switched to AA from AT to avoid their side effects (such as tardive dyskinesia) to treat schizophrenia as well as other 'psychotic' conditions [7]. Among 'psychotic-like' disorders, PI-OCD is one of the less investigated, mainly due to a relatively low prevalence and difficulty in detection of clinical features. Therefore we have a lack of available data in the literature for AA, especially for the most recently introduced drugs such as aripiprazole.

Nevertheless, available studies have demonstrated that selective SRI (SSRI) augmentation with AAs should be considered as a strategy for OCD patients who fail to show a treatment response after an 'adequate treatment', as defined by APA guidelines [8]. Additionally, in this particular patient, a history of poor clinical response to adequate SSRI therapy, as well as a persistent lack of insight and compliance, suggested treatment augmentation with AA.

The choice of aripiprazole from different AA agents was also suggested by its hypothesised mechanism of action. Aripiprazole is chemically different from other AA agents, acting as a weak stimulator of dopamine D_2 _receptors (the drug is a so-called partial agonist) with an antagonistic (inhibitory) or agonist (stimulating) activity, depending on the sensibility of the receptors and the availability of dopamine (DA). The D_2 _receptors are present both presynaptically (as autoinhibitory targets) and postsynaptically (as stimulating-heteroreceptors).

Dose-dependent effects are exerted mainly on the presynaptic D_2 _receptors with low concentrations of the antipsychotic agent, while higher dosages have the opposite effect (acting on the postsynaptic target) [9].

Aripiprazole exerts similar partial agonist effects on serotonin 1A receptors (5-HT_1A_), differently to the mechanism of other AA agents. The whole AA class share the '5-HT_2A _> D_2_' blocking mechanism [10]. Furthermore, some authors, after reviewing published and unpublished data, suggested that aripiprazole would not affect serotonin (5-HT) receptors at therapeutic doses [11].

*In vivo *studies in rats report a reduced 5-HT and an increased DA output in the medial prefrontal cortex (mPFC) and dorsal raphe nucleus compared to haloperidol, through the activation of 5-HT_1A _receptors [12]. This specific pharmacodynamic properties suggest that aripiprazole is a 'new generation' AA. Consequently, aripiprazole augmentation should lead to a greater clinical improvement of PI-OCD patients compared to patients on SSRIs. Furthermore, evidence of a DA involvement in OCD is confirmed both by animal and clinical studies [13].

In summary, in view of the clinical and pharmacodynamic aspects, the patient's mild clinical improvement appeared significant compared to the clinical response previously obtained with just SSRIs. We also hypothesised that a longer follow-up with SSRI augmentation with aripiprazole would produce a further improvement of the OCD sympthomatology.

Finally, even if caution is suggested before switching to a relatively novel treatment, we hypothesise that aripiprazole is a useful adjunctive drug in the therapy of severe PI-OCD patients. Because of paucity of data on the use of aripiprazole in PI-OCD patients, our hypothesis should be further investigated in larger patients sample with the use of systematic investigations including randomised clinical trials (RCTs).

## Competing interests

The authors declare that they have no competing interests.

## Authors' contributions

MF designed the study. FG contributed to the manuscript's drafting. CM and VV contributed to the clinical and rating evaluations durign the follow-up period. PF conceived of the study, and participated in its design and coordination. All authors read and approved the final manuscript.
